# INAVA promotes aggressiveness of papillary thyroid cancer by upregulating MMP9 expression

**DOI:** 10.1186/s13578-018-0224-4

**Published:** 2018-04-05

**Authors:** Hongyu Guan, Yan Guo, Liehua Liu, Runyi Ye, Weiwei Liang, Hai Li, Haipeng Xiao, Yanbing Li

**Affiliations:** 1 Department of Endocrinology and Diabetes Center, The First Affiliated Hospital of Sun Yat-sen University, 58 Zhongshan Road II, Guangzhou, 510080 Guangdong China; 2 Department of Thyroid and Breast Surgery, The First Affiliated Hospital of Sun Yat-sen University, Guangzhou, China

**Keywords:** INAVA, Papillary thyroid cancer, Invasion, MMP9, FGF1

## Abstract

**Background:**

Innate immunity activator (INAVA) has been shown to be elevated in lung adenocarcinoma. However, its expression pattern and function in papillary thyroid cancer (PTC) are unknown. This study aimed to identify the clinical, biological, and mechanistic impacts of INAVA on PTC.

**Methods:**

Using The Cancer Genome Atlas dataset, real time PCR, and immunohistochemistry, the expression of INAVA in PTC was analyzed. Gain- and loss-of-function assays were performed to investigate the role of INAVA in PTC cell invasion, migration, and metastasis. We explored the molecular mechanisms underlying the roles of INAVA in PTC cells using transcriptome resequencing, real time PCR, western blotting and immunohistochemistry.

**Results:**

We found that INAVA expression was significantly upregulated in PTC and was significantly associated with lymph node metastasis. Loss- and gain-of-function experiments demonstrated that INAVA promoted the aggressive phenotype of PTC cells in vitro and in vivo. Mechanistic study suggested that upregulation of INAVA resulted in elevated fibroblast growth factor 1 (FGF1), which in turn increased the expression level of matrix metalloproteinases 9 (MMP9). We further identified that the level of INAVA was positively correlated with the levels of FGF1 and MMP9 in clinical PTC specimens.

**Conclusion:**

These data establish a novel role for INAVA in promoting PTC progression and suggest that INAVA may represent a therapeutic target for the disease.

**Electronic supplementary material:**

The online version of this article (10.1186/s13578-018-0224-4) contains supplementary material, which is available to authorized users.

## Background

Thyroid cancer is the most common type of endocrine-related malignancy and shows rapidly increasing incidences during the past decades [1]. Papillary thyroid cancer (PTC) is the most common form of thyroid cancer, representing 70–80% of all thyroid cancer [2]. In most cases, patients with PTC follow an indolent course and have excellent prognosis [3]. However, extrathyroidal extension and distant metastasis are extremely important risk factors for PTC and are still major clinical challenges [4, 5]. Thus, a good understanding of the molecular alterations responsible for aggressive behavior of PTC is still imperative.

Tumor metastasis is a multistep process during which a subset of cancer cells disseminate from the site of a primary tumor and establish secondary tumors at distant sites [6]. Matrix metalloproteinases (MMPs) are proteolytic enzymes responsible for remodeling extracellular matrix (ECM) in many physiological and pathological processes [7]. Moreover, besides its role in degradation of ECM molecules, MMPs play important roles in multiple aspects of cancer progression, including tumor growth, almost all metastatic steps, and angiogenesis [8, 9]. MMP9, one of the best characterized members in MMP family, is well known to degrade ECM and is closely related to the cancer invasion and metastasis [10]. In PTC, Ahmed et al. showed that silencing of FoxM1 inhibited migration and invasion of PTC cells via decreasing the expression levels of MMP9 and MMP2 [11]. Moreover, the expression level of arachidonate 5-lipoxygenase (ALOX5) was upregulated in PTC and it promoted PTC cell invasion through increased secretion of MMP9 [12].

Innate immunity activator (INAVA, also named chromosome 1 open reading frame 106) is a protein coding gene known to be a risk locus for Crohn’s disease [13, 14]. Notably, a global analysis of chromosome 1 genes among patients with lung adenocarcinoma found that INAVA was upregulated at four stages of lung adenocarcinoma, implicating that INAVA may play important roles in cancer development and progression [15]. Nevertheless, the role of INAVA has not been investigated in malignant diseases. Here, we investigated the expression pattern of INAVA in human PTC and attempted to explore the molecular mechanisms mediating the possible oncogenic functions of INAVA.

## Methods

### Cell lines and reagents

The human PTC cell line K1 was purchased from the European Collection of Cell Cultures (ECACC, Salisbury, UK), and the TPC1 cell line was provided by Prof. Haixia Guan (China Medical University, China). All cell lines were authenticated by short tandem repeat (STR) DNA profiling and were verified to be mycoplasma-free. The cells were cultured in Dulbecco’s modified Eagle’s medium (DMEM) (Gibco, Grand Island, NY) containing 10% fetal bovine serum (FBS; Gibco), 100 U/mL penicillin (Gibco), and 100 mg/mL streptomycin (Gibco) at 37 °C in a 5% CO_2_ humidified incubator.

### Patients and tissue specimens

The 112 cases of paraffin-embedded PTC and 16 pairs of fresh PTC and their adjacent noncancerous thyroid tissues were histopathologically and clinically diagnosed at the First Affiliated Hospital of Sun Yat-sen University from 2011 to 2016. The 16 pairs of fresh tissues were collected from 16 patients, and then were frozen and stored in liquid nitrogen until used. The pathology of all specimens was confirmed by two pathologists. Informed consents from patients and ethics approval from the Institutional Research Ethics Committee were obtained.

### Immunohistochemistry (IHC)

Paraffin sections were deparaffinized with xylene, rehydrated with alcohol, subjected to antigen retrieval in sodium citrate buffer (10 mM, pH 6.0) and blocked in PBS containing 1% (wt/vol) bovine serum albumin (BSA; Sigma-Aldrich, St. Louis, MO). Then the sections were incubated with primary antibodies against INAVA and fibroblast growth factor 1 (FGF1) (Abcam, Cambridge, MA), MMP9 (Cell Signaling Technology). The IHC staining was evaluated as described in our previous study [16]. Briefly, the intensity of staining was scored as 0 (no staining), 1 (weak staining = light yellow), 2 (moderate staining = yellow brown), or 3 (strong staining = brown). The proportion of tumor cells was scored as 0 (no positive tumor cells), 1 (< 10% positive tumor cells), 2 (10–50% positive tumor cells), or 3 (> 50% positive tumor cells). The staining index (SI) was calculated by multiplying the intensity and proportion score. An SI score of ≥ 4 was regarded as tumors with high expression and ≤ 3 as tumors with low expression.

### Vectors and retroviral infection

INAVA construct was generated by sub-cloning PCR-amplified full-length human INAVA cDNA into pQCXIP (Clontech, Mountain View, CA). The pRS-INAVA-shRNAs (TR306059) were purchased from OriGene (Rockville, MD). These plasmids were transfected into PT67 cells (Clontech) using Lipofectamine 3000 (Invitrogen, San Diego, CA). After transfection, the PT67 cells were cultured for 48 h. Then, the supernatant was harvested, passed through a 0.45-μm filter and incubated with the indicated cells along with polybrene (8 μg/mL). Subsequently, stable cell lines were selected with 0.5 μg/mL of puromycin for 2 weeks [17].

### RNA extraction and real-time reverse transcription-polymerase chain reaction (RT-PCR)

Total RNA was extracted using TRIzol (Invitrogen). RT was carried out with the Moloney murine leukemia virus reverse transcriptase (MMLV-RT) (Promega, Madison, WI) using 1 μg of total RNA as the template. Real-time RT-PCR detection was performed using SYBR Premix Ex Taq (TaKaRa, Dalian, China) on a 7500 system (Applied Biosystems, Carlsbad, CA). Glyceraldehyde-3-phosphate dehydrogenase (GAPDH) was used as an internal control. The following primers were used: MMP9 forward, 5′-ACGACGTCTTCCAGTACCGA-3′ and reverse, 5′-TTGGTCCACCTGGTTCAACT-3′; FGF1 forward, 5′-TGTGGAGAGAGGTACAGCCC-3′ and reverse, 5′-AAGGTGGTGATTTCCCCTTC-3′; GAPDH forward, 5′-GACTCATGACCACAGTCCATG-3′ and reverse, 5′-AGAGGCAGGGATGATGTTCTG-3′. The relative expression levels were determined using the 2^−ΔΔCt^ method. Each sample was analyzed three times in triplicate.

### Western blotting (WB) analysis

The indicated cells were harvested and lysed using RIPA lysis buffer (Thermo Fisher Scientific, Waltham, MA). The concentration of protein was determined by the bicinchoninic acid assay (BCA; Thermo Fisher Scientific). Equal amounts of proteins were separated by sodium dodecyl sulfate–polyacrylamide gel electrophoresis (SDS-PAGE) gels and then transferred to the PVDF membranes (Roche, Indianapolis, IN). The membranes were blocked with 5% nonfat milk and then incubated with the primary antibodies: anti-INAVA antibody and anti-FGF1 antibody (Abcam), anti-MMP9 antibody (Cell Signaling Technology), and anti-α-tubulin antibody (Sigma-Aldrich). The bands were detected using enhanced chemiluminescence (ECL) detection kit (Pierce, Rockford, IL) according to the manufacturer’s instructions.

### Transwell assay

The invasion and migration assays were performed using Transwell chambers (Millipore, Bedford, MA) pre-coated with or without Matrigel (BD, Bedford, MA) on the upper side in 24-well plates. A total of 2 × 10^4^ cells in 200 μL of serum-free DMEM medium were added to the upper chamber and 500 μL of DMEM medium containing 10% FBS were added to the lower chamber. Following 24 h incubation, the cells on the upper surface of the chambers were removed with cotton swabs. Then the migrated and invaded cells on the lower surface were stained with 0.1% crystal violet and counted under a microscope. Each sample was analyzed three times in triplicate.

### Wound-healing assay

The cell migration activity was examined using wound-healing assay. Briefly, the indicated cells were seeded in 6-well plates and grown to reach 90–95% confluence. Then the wounds were created by scratching the cell monolayer with a micropipette tip. Cells were allowed to migrate for 18 h under serum-reduced medium. The wounds were observed and photographed under a microscope. The Image J software (National Institutes of Health, Bethesda, Maryland) was used to determine the distance between the wound edges.

### In vivo metastasis assay

For the in vivo metastasis assay, equal amounts of (6 × 10^6^) the luciferase-expressing K1-INAVA-Luc or K1-Vector-Luc cells were injected through the tail vein of 6 weeks old BALB/c nude mice (5 mice per group). Bioluminescence imaging was performed using the Xenogen IVIS Spectrum Imaging System (Caliper Life Sciences, Hopkinton, MA). Mice were injected i.p. with 150 mg/kg luciferin 15 min prior to imaging. Images were taken and analyzed using Spectrum Living Image 4.0 Software (Caliper Life Sciences). Sixty days post-injection, the mice were euthanized. The lungs were excised, embedded with paraffin and then stained with hematoxylin and eosin (HE) [18]. Microscopic evaluation was carried out using the BX51 Olympus microscope (Olympus, Tokyo, Japan) and photographs were obtained. Metastasis area was calculated using Image J software in HE stained sections.

### RNA sequencing

Transcriptome resequencing was performed by the Vazyme Biotech Co., Ltd, (Nanjing, China). Briefly, the transcriptome library for sequencing was generated using VAHTSTM mRNA-seq v2 Library Prep Kit for Illumina^®^ (Vazyme Biotech Co.) following the manufacturer’s recommendations. The clustering of the index-coded samples was performed using VAHTS RNA Adapters set1/set2 for Illumina^®^ (Vazyme Biotech Co.) according to the manufacturer’s instructions. After clustering, the libraries were sequenced on Illumina Hiseq X Ten platform using paired-end module. Then, bioinformatics analysis was performed.

### Accession numbers

The transcriptome resequencing data described herein have been deposited in the Gene Expression Omnibus (GEO) database and are accessible through GEO series accession number GSE100392.

### Gelatin zymography analysis

Zymography assay was performed to examine the MMP activity. In brief, indicated cells were cultured in serum-free DMEM medium for 48 h before the culture media were collected and centrifuged. Equal amounts of supernatants were added with equal amounts of 2× Zymogram sample buffer (Bio-Rad, Hercules, CA) and subjected to Ready Gel Zymogram gels (Bio-Rad) under nonreducing condition. After electrophoresis, the gel was renatured in renaturation buffer (Bio-Rad) and developed in development buffer (Bio-Rad). Finally, the gels were stained with 0.5% Coomassie brilliant blue R-250 and photographed.

### Transient transfection

The siRNAs for MMP9 (MMP9-si1, 5′-CCACCACAACATCACCTAT-3′; MMP9-si2, 5′-GCATAAGGACGACGTGAAT-3′) were designed and synthesized by RiboBio Co. (Guangzhou, China). The siRNAs for FGF1 were purchased from OriGene (SR301569). Transfection was performed using Lipofectamine 3000 (Invitrogen) in accordance with the manufacturer’s instructions.

### Enzyme linked immunosorbent assay (ELISA)

The levels of FGF1 in cell culture supernatants were assessed using a human FGF1 ELISA kit (Thermo Fisher Scientific) according to the manufacturer’s protocol.

### Bioinformatic analysis

RNAseqV2 data of 59 pairs of PTC tissues versus paired adjacent noncancerous thyroid tissues and 496 cases of PTC tissues were mined from The Cancer Genome Atlas (TCGA) (https://cancergenome.nih.gov/) using UCSC Xena (http://xena.ucsc.edu/getting-started/).

### Statistical analysis

All statistical analyses were performed using the SPSS17.0 software (SPSS Inc., Chicago, IL). The data were expressed as mean ± standard deviation (SD). Student *t* test was performed to compare the differences between two groups. P < 0.05 was considered as statistically significant.

## Results

### INAVA is upregulated in PTC and associated with clinicopathologic characteristics of patients

**Fig. 1:**
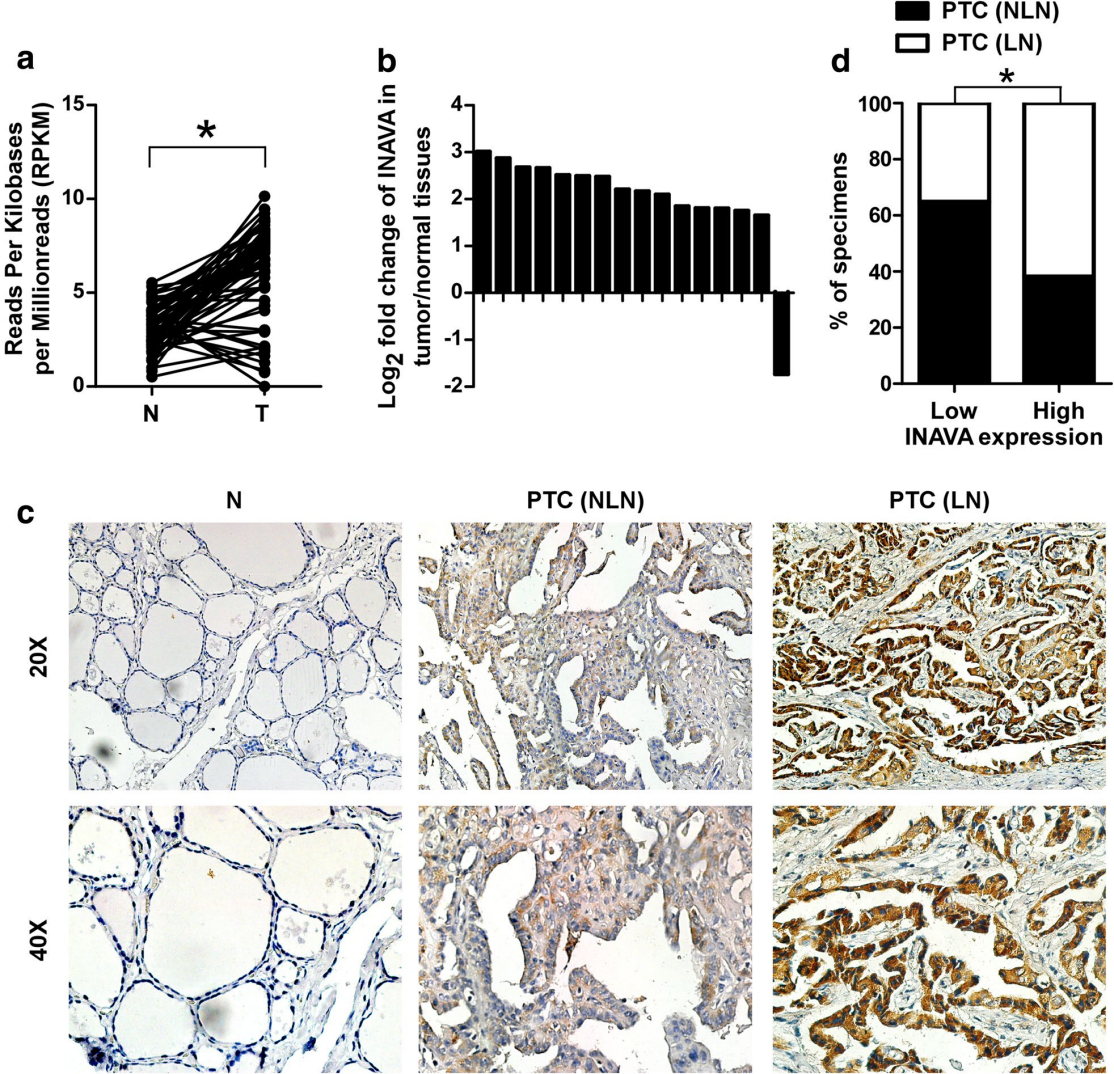
INAVA is upregulated in PTC and is associated with LN metastasis. **a** The expression of INAVA in 59 pairs of primary PTC (T) versus their paired non-cancerous thyroid tissues (N) using RNAseq data sets deposited on TCGA. **b** The expression of INAVA in 16 paired T and N was assessed by real time RT-PCR. GAPDH was used as a house keeping gene control. **c** Representative images of IHC assays on INAVA expression in PTC specimens from the studied cohort. *NLN* with no lymph node metastasis, *LN* with lymph node metastasis. **d** Percentage of specimens showing low (n = 57) or high (n = 55) INAVA expression in relation to the LN metastasis. *P < 0.05

Initially, we assessed the expression of INAVA in PTC. To this end, we analyzed the INAVA expression in 59 pairs of PTC specimens and their corresponding adjacent noncancerous thyroid tissues using thyroid cancer RNAseq data deposited on TCGA. As shown in Fig. 1a, INAVA expression level was upregulated in most (49/59) PTC tissues as compared with their paired adjacent noncancerous thyroid tissues. Next, we collected 16 pairs of PTC and adjacent noncancerous tissues and assessed the expression of INAVA using real time RT-PCR. As shown in Fig. 1b, INAVA expression was elevated in tumor tissues. We further analyzed whether the expression of INAVA is correlated with clinical variables. As shown in Table 1, using RNAseq data deposited on TCGA (496 cases of PTC with clinical information), we found that INAVA expression was significantly associated with N classification (*P *< 0.001), tumor-node-metastasis (TNM) staging (*P *= 0.010), and T classification (*P *= 0.017). We further performed IHC to detect the expression of INAVA in 112 cases of PTC specimens. Consistent with the results obtained by analyzing TCGA dataset, the expression level of INAVA in PTC was significantly increased as compared with that in normal thyroid tissues, and also significantly associated with TNM staging (*P *= 0.007) and N classification (*P* = 0.005) (Table 2). INAVA expression significantly increased in PTC with LN metastasis (LN) as compared with that in PTC with no LN metastasis (NLN) (Fig. 1c, d). Taken together, these data indicate that INAVA expression is elevated in PTC and associated with clinicopathologic features of patients with the disease.

**Table 1:** Association between INAVA expression and clinicopathologic parameters in PTC patients using TCGA dataset

Clinicopathologic variables (n)	INAVA		*P* value
	Low	High	
Age (years)			
< 45 (331)	156	175	0.070
≥ 45 (165)	92	73	
Gender			
Male (131)	62	69	0.476
Female (365)	186	179	
TNM stage			
I and II (329)	178	151	0.010
III and IV (165)	69	96	
Unknown (2)			
T classification			
T1 and T2 (307)	167	140	0.017
T3 and T4 (187)	81	106	
Unknown (2)			
Lymph node metastasis			
No (227)	135	92	< 0.001
Yes (219)	83	136	
Unknown (50)

**Table 2:** Association between INAVA expression and clinicopathologic parameters in PTC patients

Clinicopathologic variables (n)	INAVA		*P* value
	Low	High	
Age (years)			
< 45 (59)	32	27	0.455
≥ 45 (53)	25	28	
Gender			
Male (23)	12	11	0.890
Female (89)	45	44	
TNM stage			
I and II (73)	44	29	0.007
III and IV (39)	13	26	
T classification			
T1 and T2 (69)	40	29	0.058
T3 and T4 (43)	17	26	
Lymph node metastasis			
No (58)	37	21	0.005
Yes (54)	20	34	

### Overexpression of INAVA promotes PTC cells invasion, migration and metastasis

**Fig. 2:**
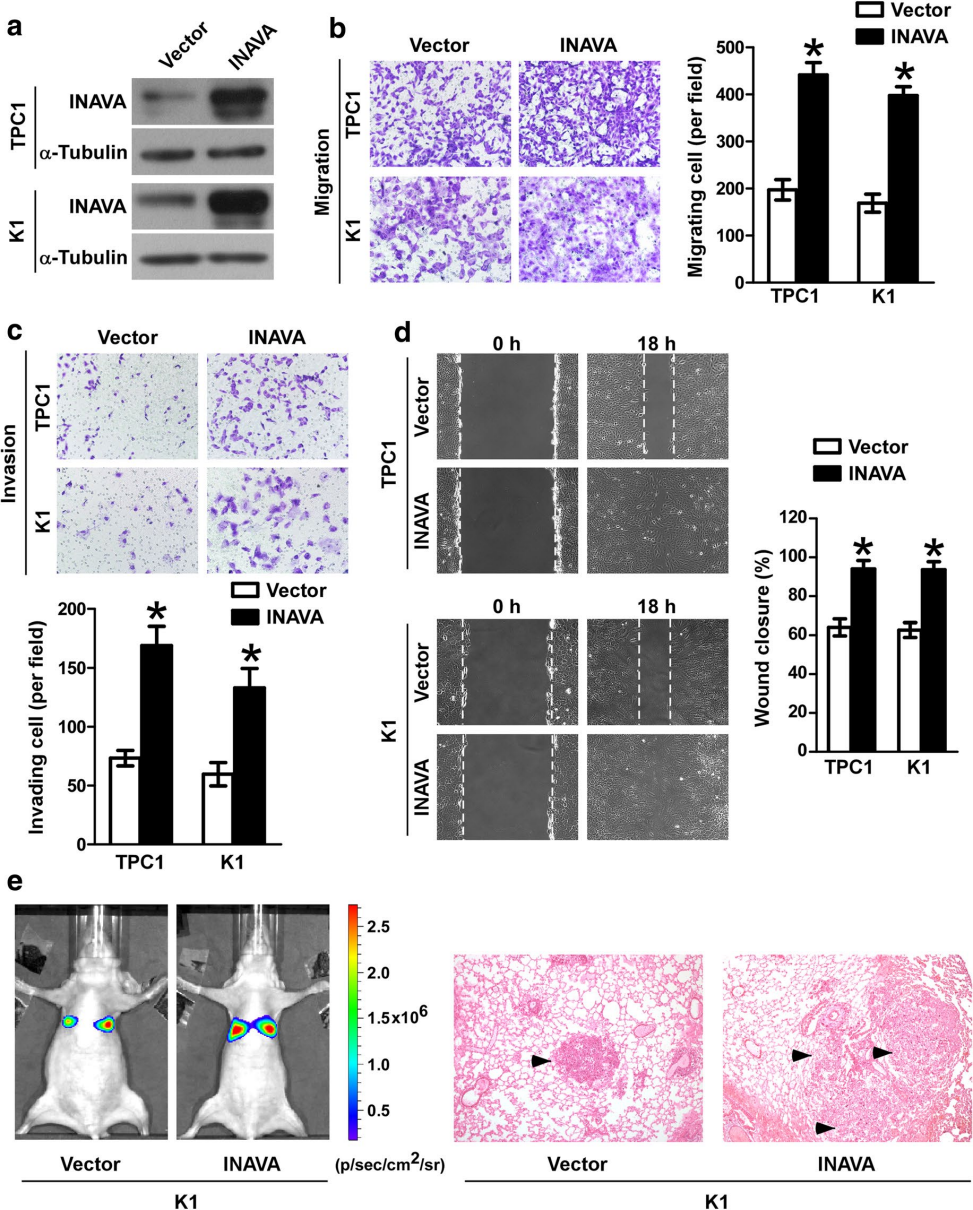
Overexpression of INAVA promotes cell invasion, migration and metastasis. **a** Overexpression of INAVA in PTC cell lines (TPC1 and K1) was assessed by WB. α-Tubulin was used as a loading control. **b** Representative images (left) and quantification (right) of transwell migration assays in TPC1, TPC1-INAVA, K1, and K1-INAVA cells. **c** Representative images (upper) and quantification (lower) of Transwell invasion assays in indicated cells. **d** Scratch wound healing assays were performed in TPC1-Vector, TPC1-INAVA, K1-Vector, and K1-INAVA cells (left). Quantification analyses for the wound healing assays were shown (right). **e** Representative bioluminescence images in mice with tail vein injection of the K1-Vector and K1-INAVA cells (left). Representative histopathology of lung metastasis developed in the indicated animals stained with HE (right). For **b**–**d**, data are quantified as mean ± SD of 3 independent experiments in the bar graphs. *P < 0.05

Given the association of INAVA with more aggressive PTC, we next explored whether the function of INAVA in PTC could explain this association. We ectopically overexpressed INAVA in two PTC cell lines (TPC1 and K1) using empty vector as a control to determine its effect on the aggressive phenotype of the cells (Fig. 2a). As shown in Fig. 2b, c, in vitro cell invasion and migration assays showed that overexpression of INAVA significantly enhanced both the invasion and migration abilities of TPC1 and K1 cells. Scratch wound healing assay was performed to assess the effect of INAVA on cellular migration and the results showed that overexpression of INAVA significantly promoted wound closure in TPC1 and K1 cells (Fig. 2d). In an in vivo experimental metastasis assay, overexpression of INAVA promoted mouse lung colonization by tail vein-injected K1 cells (Fig. 2e and Additional file 1: Figure S1a).

### Knockdown of INAVA inhibits PTC cells invasion, migration and metastasis

**Fig. 3:**
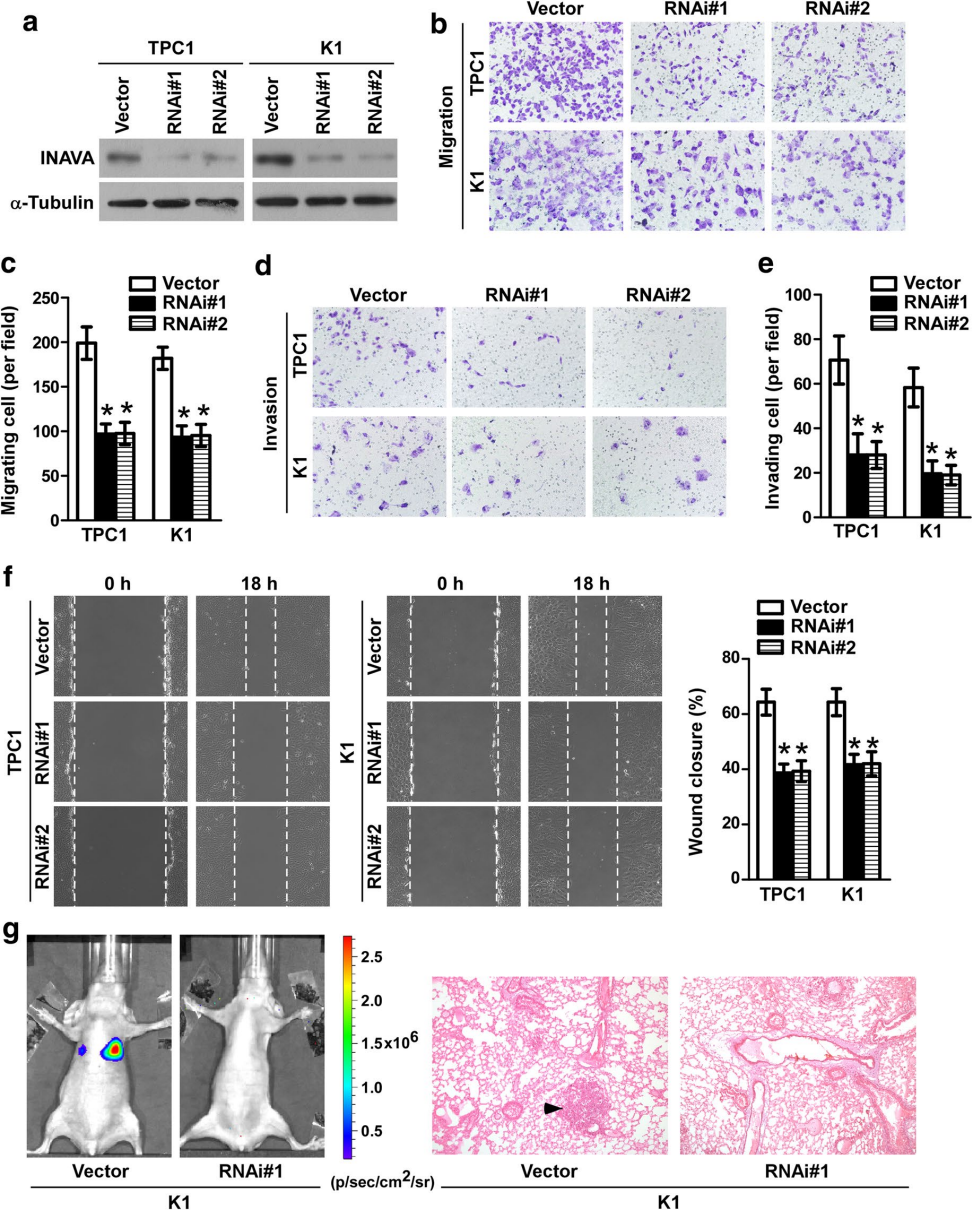
Silencing of INAVA inhibits cell invasion, migration and metastasis. **a** Protein expression levels of INAVA in INAVA-silencing and vector-control cells were analyzed by WB. α-Tubulin was used as a loading control. Representative images (**b**) and quantification (**c**) of transwell migration assays in indicated cells. Representative images (**d**) and quantification (**e**) of transwell invasion assays in indicated cells. **f** Scratch wound healing assays were performed in indicated cells (left) and quantification analyses for the assays were shown (right). **g** Representative bioluminescence images in mice with tail vein injection of the indicated cells (left) and representative histopathology of lung metastasis stained with HE was shown (right). For **b**–**f**, data are quantified as mean ± SD of 3 independent experiments in the bar graphs. *P < 0.05

We next established pools of TPC1 and K1 cell lines with stable depletion of INAVA using retroviral-based shRNA vectors (Fig. 3a). As shown in Fig. 3b–e, Transwell assay with or without Matrigel coating showed that silencing of INAVA significantly decreased the invasiveness and migration of the TPC1 and K1 cell lines. Moreover, wound healing assay showed that knockdown of INAVA inhibited the migratory speed of TPC1 and K1 cells in comparison with that of the vector controls (Fig. 3f). In addition, knockdown of INAVA in K1 cells significantly decreased pulmonary metastatic nodule development (Fig. 3g and Additional file 1: Figure S1b). These data, together with results of Fig. 2, suggest that INAVA greatly contributes to the development of PTC invasion and metastasis.

### MMP9 is the key molecule mediating INAVA-induced invasion and migration ability of PTC cells

**Fig. 4:**
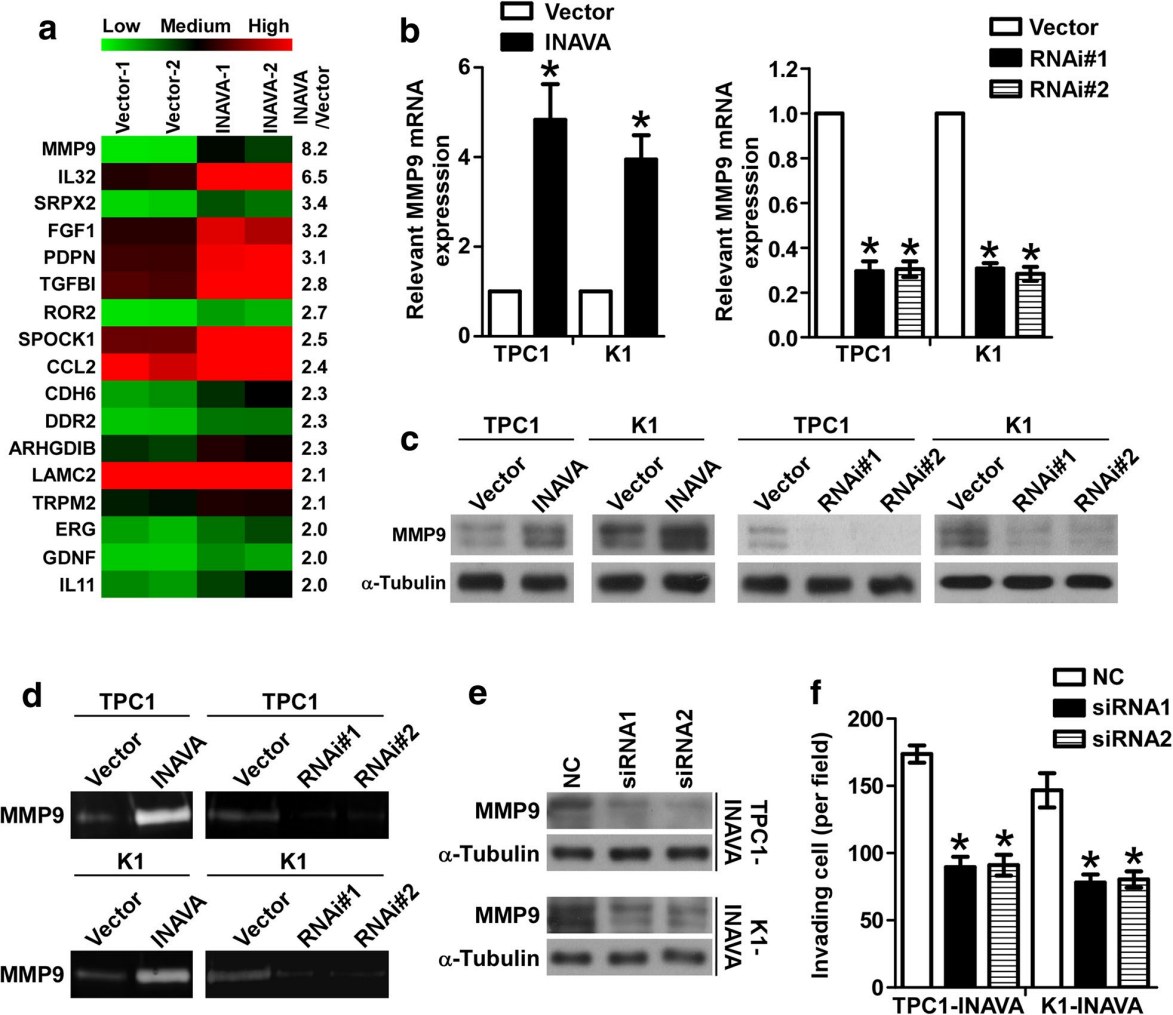
The effect of INAVA on PTC cells is mediated by MMP9. **a** Transcriptome resequencing was performed on TPC1-Vector and TPC1-INAVA cells and mRNA expression of the indicated genes related to invasion/metastasis is shown. **b** Altered cellular mRNA levels of MMP9 in the indicated cells were detected by real time RT-PCR assays. GAPDH served as a house keeping gene control. **c** WB assays were conducted to analyze the protein levels of MMP9 in response to deregulated INAVA expression in TPC1 and K1 cells. α-Tubulin acted as a loading control. **d** MMP9 activities in indicated cells were analyzed using gelatin zymography. Knockdown of MMP9 in INAVA overexpressing cells (**e**), attenuated phenotypic characteristics induced by INAVA overexpression (**f**). For **b**, **f**, data represents mean ± SD of 3 independent experiments in the bar graphs. *P < 0.05

To explore the molecular mechanisms by which INAVA regulates the invasion and metastasis ability of PTC cells, transcriptome resequencing was performed on INAVA-overexpressing TPC1 cells and vector control cells. Notably, many invasion/metastasis related genes were significantly increased in INAVA overexpressing TPC1 cells as compared with that in vector control (Fig. 4a). Importantly, among these genes, MMP9 was the most elevated gene in response to ectopic overexpression of INAVA. Next, real time RT-PCR assays were performed to confirm the results of deep sequencing analysis. As shown in Fig. 4b, the MMP9 mRNA expression levels were significantly increased and decreased in INAVA overexpressing and knockdown cell lines, respectively, suggesting that transcriptional efficiency of MMP9 is regulated by INAVA in PTC cells. The protein levels of MMP9 in indicated cells were in line with the changes of mRNA (Fig. 4c). As shown in Fig. 4d, the results of gelatin zymography showed that the secretion of MMP9 into culture medium was significantly increased in cells overexpressing INAVA and was significantly decreased in cells with INAVA knockdown. Moreover, knockdown of MMP9 using specific siRNAs in INAVA overexpressing TPC1 and K1 cells (Fig. 4e) suppressed the phenotype caused by INAVA overexpression (Fig. 4f). These results indicate that MMP9 plays a vital role in INAVA-induced cell invasion and migration.

### FGF1 mediates the regulation of INAVA on MMP9

**Fig. 5:**
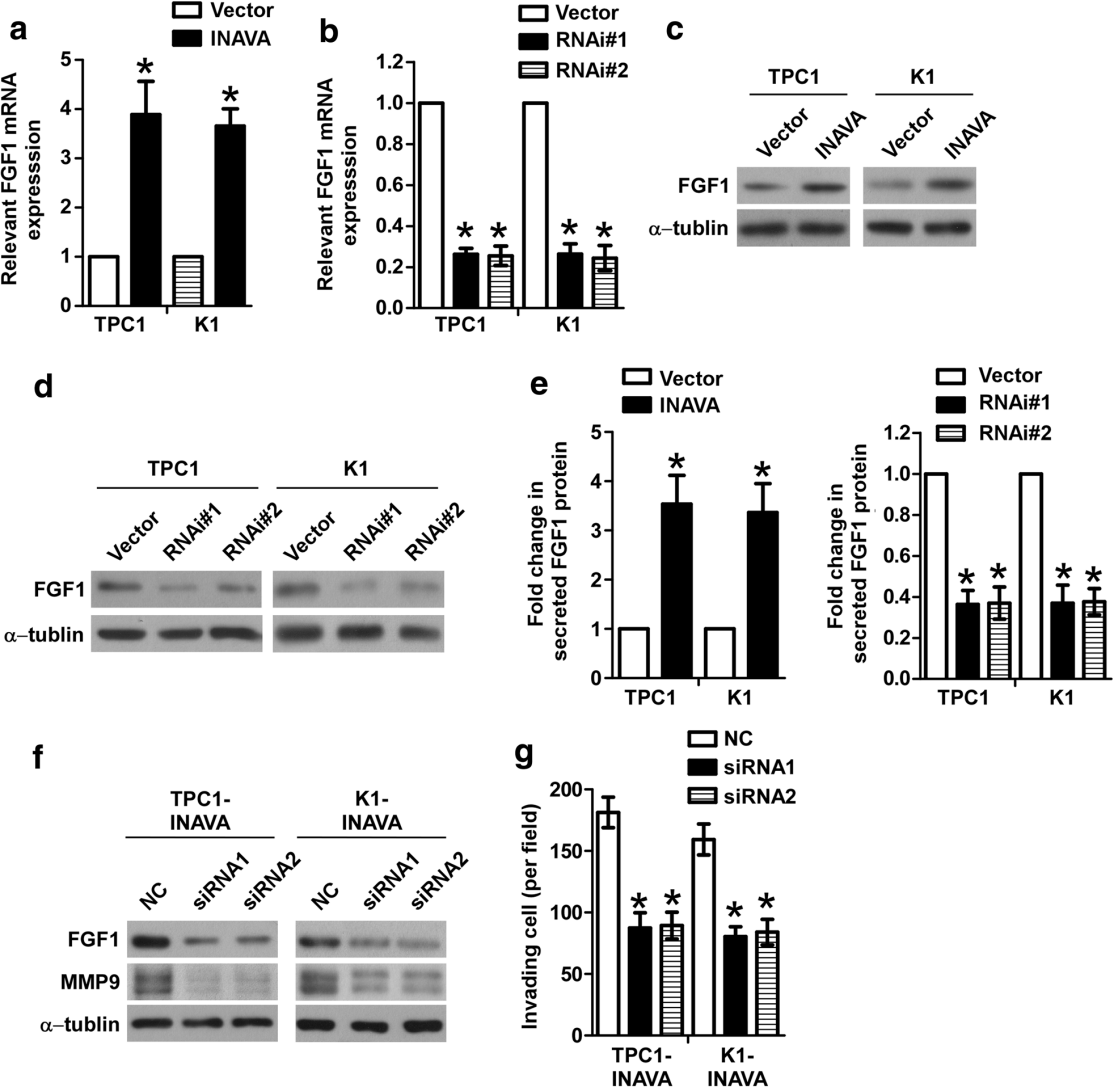
INAVA modulates MMP9 via regulating FGF1. mRNA levels of FGF1 were examined in INAVA-overexpressing (**a**) or -knockdown cells (**b**). **c**, **d** Protein levels of FGF1 were assessed in indicated cells. **e** ELISA assays were performed to assess the effect of INAVA on FGF1 release in TPC1 and K1 cells. **f** Knockdown of FGF1 by siRNAs in INAVA-overexpressing cells reversed increased level of MMP9. **g** Invasiveness caused by INAVA was abrogated by silencing FGF1 expression in INAVA-overexpressing cells. For **a**, **b**, **e**, **g**, results derived from three independent experiments are expressed as mean ± SD. *P < 0.05

Given that MMP9 has been demonstrated to be regulated by FGF1 [19], together with that the results of deep sequencing analysis showed FGF1 was significantly upregulated in INAVA-overexpressing cells (Fig. 4a), we hypothesized that FGF1 was involved in the role of INAVA on regulation of MMP9. As expected, the mRNA and protein levels of FGF1 were elevated by overexpression of INAVA but were decreased by depletion of INAVA in PTC cells (Fig. 5a–d). Consistently, ELISA assays showed that INAVA-overexpressing cells secreted increased amount of FGF1, whereas INAVA silencing reduced the secretion of FGF1 (Fig. 5e). To further confirm the involvement of FGF1 in INAVA-mediated effects, the specific siRNAs were used to knockdown FGF1 in INAVA overexpressing cells. As shown in Fig. 5f, the upregulation of MMP9 induced by INAVA were inhibited by FGF1 silencing. As anticipated, invasive phenotype caused by INAVA overexpression was also suppressed by depletion of FGF1 (Fig. 5g). Taken together, these data indicate FGF1 play a vital role in INAVA-induced MMP9 expression and cell invasion.

### INAVA expression correlates with FGF1 and MMP9 expression in primary PTC

**Fig. 6:**
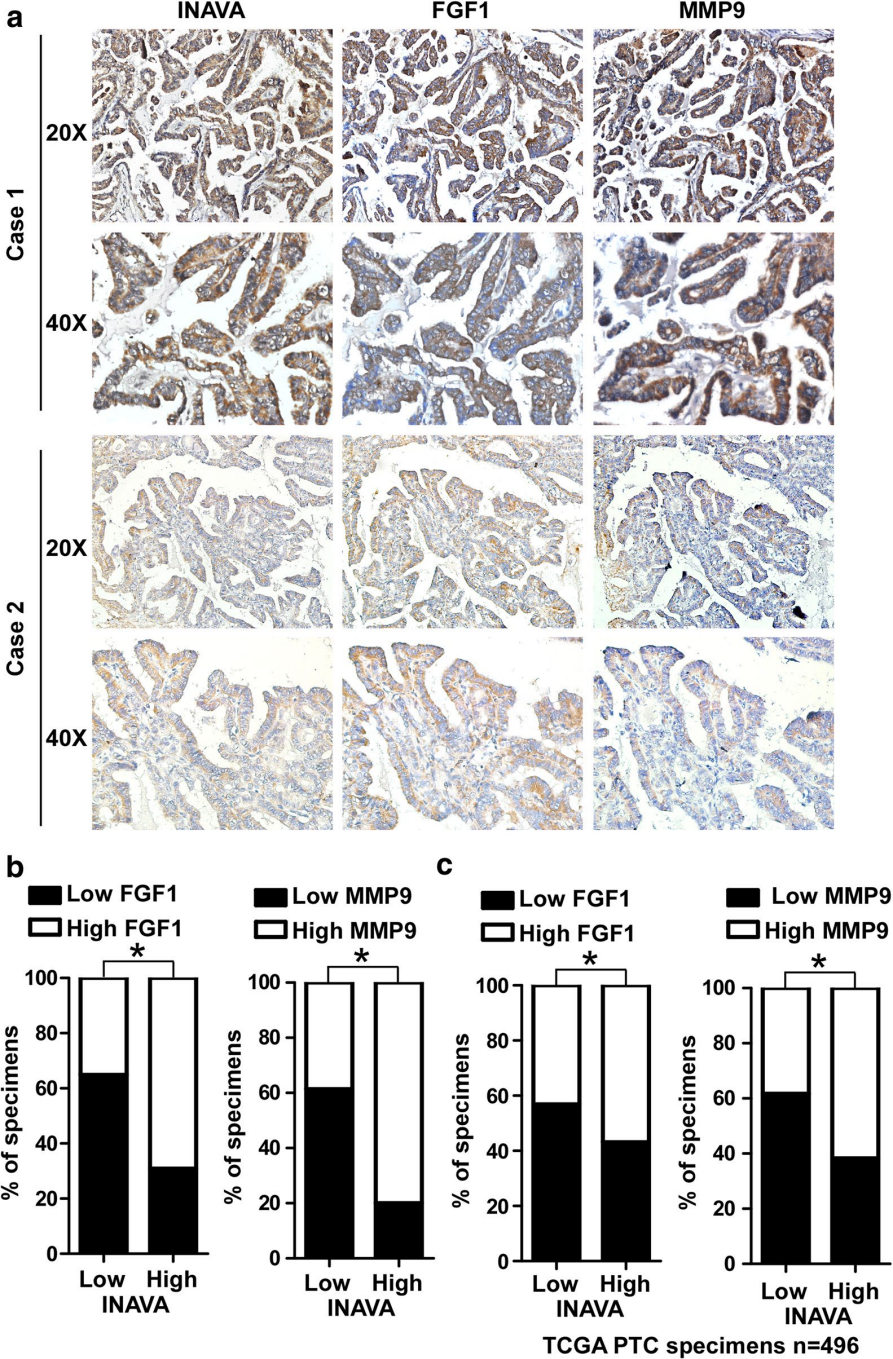
INAVA positively correlates with FGF1 and MMP9 in PTC clinical specimens. **a** The expression of INAVA is associated with FGF1 and MMP9 in clinical PTC specimens. Two representative cases are shown. **b** Percentage of cases showing low (n = 57) or high (n = 55) INAVA expression in relation to the FGF1 and MMP9 expression levels. **c** The association between INAVA and FGF1 as well as MMP9 in 496 cases of PTC specimens using RNAseq data deposited on TCGA. The expression level of INAVA was divided into low (n = 248) and high (n = 248) groups according to median distribution. For **b**, **c**, *P < 0.05

To evaluate the correlations between INAVA expression and the levels of FGF1 and MMP9 in clinical specimens, IHC analyses were performed. As shown in Fig. 6a, b, 64.9% (37 cases) and 61.4% (35 cases) of samples with low INAVA expression (57 cases), respectively, exhibited low levels of FGF1 and MMP9, whereas 30.9% (17 cases) and 20.0% (11 cases) of samples with high INAVA expression (55 cases) showed low expression of FGF1 and MMP9, respectively (*P* < 0.05). Furthermore, we sorted patient cohort into low and high INAVA expression using RNAseq data mined from TCGA (INAVA low and high, n = 248 respectively). As shown in Fig. 6c, INAVA expression significantly correlated with the levels of FGF1 and MMP9 in PTC specimens. These results suggest that INAVA overexpression in PTC is associated with FGF1–MMP9 axis.

## Discussion

The results of this study provided strong evidence supporting the oncogenic roles of INAVA in PTC. First, the expression of INAVA is significantly elevated in PTC specimens as compared with that in the adjacent non-tumorous thyroid specimens. Second, the increased expression of INAVA is associated with clinicopathologic parameters in PTC patients. Third, INAVA promotes the aggressive phenotypes of PTC cells in vitro and in vivo. Fourth, INAVA regulates FGF1–MMP9 axis, thereby contributing to aggressive phenotypes of PTC.

Although the expression of INAVA has been reported to be elevated in lung adenocarcinoma [15], its roles in cancer development and progression are largely unknown. While the molecular mechanism underlying the observed INAVA upregulation in PTC still need to be further investigated, our current data strongly showed that INAVA was significantly upregulated in PTC and was remarkably associated with cervical lymph metastasis by analyzing the TCGA data and our clinical cohorts. We therefore explored its pro-invasive and pro-metastatic functions in PTC cells by a series of in vitro and in vivo experiments. Our results showed that overexpression of INAVA led to enhancement of the invasive and metastatic capability of TPC1 and K1 cells. On contrary, INAVA silencing in PTC cells showed significantly inhibitory effects on invasion and metastasis. Our data demonstrate that INAVA plays an important role in the progression of malignant disease.

MMP9 degrades type IV collagen and plays important roles in various pathophysiological processes, such as wound healing, tumor invasiveness and progression [20, 21]. It has been demonstrated that MMP9 play important roles in the development and progression of thyroid cancer. For instance, the expression of MMP9 was significantly up-regulated in PTC and was correlated with clinicopathological parameters of the disease [22–24]. MMP9 plays an important role in astrocyte elevated gene-1 induced PTC progression and metastasis [25]. Further studies that aim at investigating the regulatory mechanisms underlying the MMP9 in PTC will improve our understanding of etiology of the disease. One of the key finding of the current study is represented by the identification of a new signaling cascade in PTC cells. Elevated INAVA upregulates the expression and secretion of FGF1, which subsequently results in upregulation of MMP9 and aggressive phenotypes of PTC cells. The data derived from current mechanistic study provide an explanation for the observed association between INAVA overexpression and cervical lymph metastasis in PTC. Of note, it would also be interesting to explore whether the same chain reaction also presents in other cancer types with INAVA overexpression.

Although most thyroid cancers of follicular epithelial derivation are well differentiated and well behaved, a small subset of thyroid cancers behave aggressively via genetic and epigenetic alterations [26–28]. PTC is known for its early spread to regional lymph nodes and growing evidence has demonstrated that central lymph node metastasis is adversely associated with survival [29, 30]. Distant metastasis occurs in only < 1% of thyroid cancer, but strongly associates with poor clinical outcomes [31–33]. Delineating the molecular aspects of the LN metastasis and distant metastasis in PTC will lead to a better understanding of the disease. Our finding provides new insights into the molecular mechanisms underlying the aggressive behavior of PTC by identifying INAVA as a novel promoter of tumor cell invasion and metastasis.

FGF1, an oncogene, has been demonstrated to regulate many cellular processes, including cell invasion, proliferation, and survival [34, 35]. FGF1 is upregulated in various tumors and is involved in progression of malignances [36–38]. The elevated expression of FGF1 has been found in PTC [39]. To delineate the regulatory mechanisms of deregulation of FGF1 in cancers is of great interest. Our data obtained in vitro using TPC1 and K1 cell lines showed that overexpression of INAVA enhanced the expression and secretion of FGF1, whereas INAVA silencing inhibited FGF1 expression and secretion. In addition, our clinical findings demonstrated a strong correlation between INAVA and FGF1 expression. Our data provide new insights into the current understanding on the regulatory networks that control the expression of FGF1 in malignant diseases.

FGF1 can be released into the extracellular environment via unclear mechanisms, by doing this, it acts as a paracrine or autocrine factor to activate specific cell surface receptors [40]. There are two possible sources of FGF1 within epithelial cancers, namely, the cancer cells or surrounding stromal cells. FGF1 may be secreted from either of these two sources and acts in a paracrine and/or autocrine manner [41]. Secreted FGF1 exerts its biological function via binding to, and activation of, fibroblast growth factor receptors (FGFRs) with intrinsic tyrosine kinase activity at the cellular surface and thereby triggering intracellular signaling cascades, including mitogen-activated protein kinase (MAPK), nuclear factor-kappa B (NF-κB), and phospholipase Cγ (PLCγ) signaling [19, 42, 43]. Of specific note, MAPK and NF-κB pathways are well known for regulating the expression of MMP9 [44, 45]. In current study, we found that INAVA increased the expression as well as the secretion of FGF1 in PTC cells. It is likely that MAPK and NF-κB pathways might be involved in INAVA-induced MMP9 expression. Another interesting issue need to be further investigated is whether the FGF1 secreted by the tumor cell may act in a paracrine manner to affect the surrounding stromal cells, which in turn facilitates the development and progression of PTC.

## Conclusion

In summary, our data indicate that INAVA expression is elevated in PTC. Our data also show that INAVA expression is associated with aggressive phenotypes of PTC. We demonstrate that INAVA overexpression enhances invasion and metastasis via upregulation of MMP9. In addition, shRNA-mediated knockdown experiments further confirm the pro-invasive and pro-metastatic roles of INAVA in PTC. Furthermore, we show that the upregulation of MMP9 induced by INAVA is mediated by elevated FGF1. Taken as a whole, our data reveal a novel molecular mechanism that involves the aggressive phenotypes of PTC and may prove clinically useful for developing a new therapeutic target for PTC invasion and metastasis.

## Additional file

**Additional file 1: Figure S1.** INAVA regulates PTC cell metastasis. Metastasis area was calculated using Image J software in HE stained sections.

## Abbreviations

ALOX5: arachidonate 5-lipoxygenase
BCA: bicinchoninic acid assay
DMEM: Dulbecco’s modified Eagle’s medium
ECACC: European Collection of Cell Cultures
ECM: extracellular matrix
ECL: enhanced chemiluminescence
ELISA: enzyme linked immunosorbent assay
FGF1: fibroblast growth factor 1
GAPDH: glyceraldehyde-3-phosphate dehydrogenase
GEO: gene expression omnibus
HE: hematoxylin and eosin
IHC: immunohistochemistry
INAVA: innate immunity activator
LN: lymph node
NLN: with no lymph node metastasis
MMPs: matrix metalloproteinases
PTC: papillary thyroid cancer
RT-PCR: reverse transcription-polymerase chain reaction
SDS-PAGE: sodium dodecyl sulfate–polyacrylamide gel electrophoresis
STR: short tandem repeat
TCGA: The Cancer Genome Atlas
TNM: tumor-node-metastasis
WB: western blotting
