# Supplementary figures and images for: INAVA promotes aggressiveness of papillary thyroid cancer by upregulating MMP9 expression
Source: Cell Biosci. 2018 Apr 5;8:26. doi: 10.1186/s13578-018-0224-4 (PMC5887255; doi:10.1186/s13578-018-0224-4)

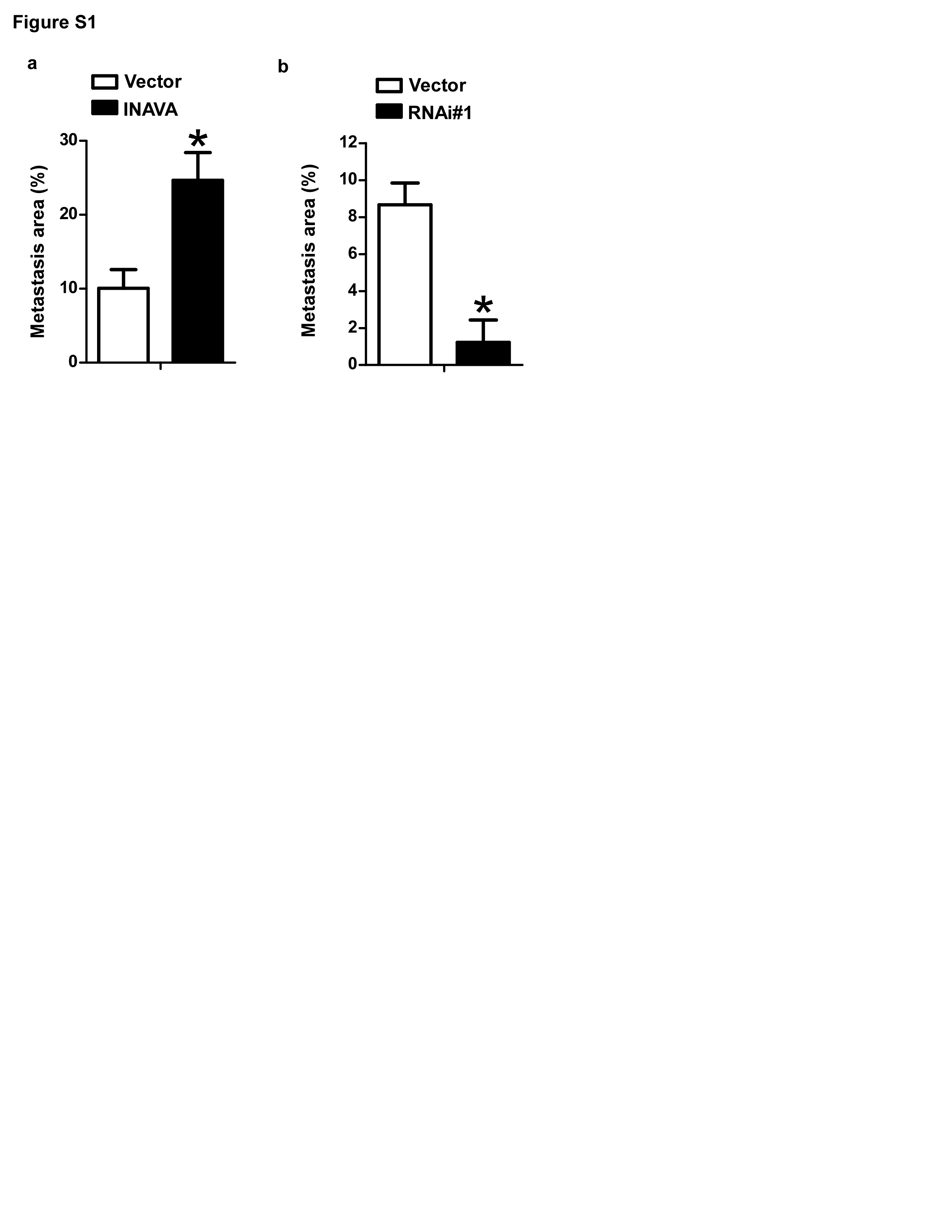

Supplement: Supplementary file 1 — Additional file 1: Figure S1. INAVA regulates PTC cell metastasis. Metastasis area was calculated using Image J software in HE stained sections. [file 13578_2018_224_MOESM1_ESM.tif]
